# Inhibition of the K^+^ Channel K_Ca_3.1 Reduces TGF-β1-Induced Premature Senescence, Myofibroblast Phenotype Transition and Proliferation of Mesangial Cells

**DOI:** 10.1371/journal.pone.0087410

**Published:** 2014-01-28

**Authors:** Rong-Guo Fu, Tao Zhang, Li Wang, Yan Du, Li-Ning Jia, Jing-Jing Hou, Gang-Lian Yao, Xiao-Dan Liu, Lei Zhang, Ling Chen, Bao-Song Gui, Rong-Liang Xue

**Affiliations:** 1 Department of Nephrology, Second Affiliated Hospital, School of Medicine, Xi'an Jiaotong University, Xi'an, Shaanxi Province, P.R. China; 2 School of Medicine, Xi'an Jiaotong University, Xi'an, Shaanxi Province, P.R. China; 3 Cadre's ward, Second Affiliated Hospital, School of Medicine, Xi'an Jiaotong University, Xi'an, P.R. China; 4 Medical Laboratory, Second Affiliated Hospital, School of Medicine, Xi'an Jiaotong University, Xi'an, P.R. China; 5 Department of Oncology, First Affiliated Hospital, School of Medicine, Xi'an Jiaotong University, Xi'an, Shaanxi Province, P.R. China; 6 Department of Anesthesia, Second Affiliated Hospital, School of Medicine, Xi'an Jiaotong University, Xi'an, Shaanxi Province, P.R. China; Institut national de la santé et de la recherche médicale (INSERM), France

## Abstract

**Objective:**

K_Ca_3.1 channel participates in many important cellular functions. This study planned to investigate the potential involvement of K_Ca_3.1 channel in premature senescence, myofibroblast phenotype transition and proliferation of mesangial cells.

**Methods & Materials:**

Rat mesangial cells were cultured together with TGF-β1 (2 ng/ml) and TGF-β1 (2 ng/ml) + TRAM-34 (16 nM) separately for specified times from 0 min to 60 min. The cells without treatment served as controls. The location of K_Ca_3.1 channels in mesangial cells was determined with Confocal laser microscope, the cell cycle of mesangial cells was assessed with flow cytometry, the protein and mRNA expression of K_Ca_3.1, α-smooth muscle actin (α-SMA) and fibroblast-specific protein-1 (FSP-1) were detected with Western blot and RT-PCR. One-way analysis of variance (ANOVA) and Student-Newman-Keuls-q test (SNK-q) were used to do statistical analysis. Statistical significance was considered at P<0.05.

**Results:**

K_ca_3.1 channels were located in the cell membranes and/or in the cytoplasm of mesangial cells. The percentage of cells in G_0_-G_1_ phase and the expression of K_ca_3.1, α-SMA and FSP-1 were elevated under the induction of TGF-β1 when compared to the control and decreased under the induction of TGF-β1+TRAM-34 when compared to the TGF-β1 induced (P<0.05 or P<0.01).

**Conclusion:**

Targeted disruption of K_Ca_3.1 inhibits TGF-β1-induced premature aging, myofibroblast-like phenotype transdifferentiation and proliferation of mesangial cells.

## Introduction

Mesangial cells are specialized smooth muscle cells around tiny blood vessels, or capillaries, in the kidney. They account for 30%∼40% of intrinsic glomerular cell totals and help regulate the filtration process of blood while providing support for the glomerular structure [1]. It has been proposed that premature senescence and myofibroblast phenotype transdifferentiation of mesangial cells contributes to the development and deterioration of glomerulosclerosis [2] and early control of phenotypic change and proliferation of mesangial cells has great importance to the prevention of glomerulosclerosis [3], [4].

The intermediate-conductance Ca(2^+^)-activated K(^+^) channel (K_Ca_3.1) is highly sensitive to intracellular Ca(2^+^), and its open probability can be sharply elevated with the increase of intracellular concentration of Ca(2^+^) [5], [6]. Normally the K_Ca_3.1 channel is in a resting state and hardly open. Under pathological conditions, however, a small amount of calcium influx may immediately activate a large number of K_Ca_3.1 channels, and the resulting huge driving force accelerates Ca(2^+^) influx, causing hypertrophy and phenotypic transition [7]–[9]. The K_Ca_3.1 has also been suggested to promote mitogenesis in several cell types and contribute to renal fibroblast proliferation and development of tubulointerstitial fibrosis in the kidney [10]. However, the potential involvement of K_Ca_3.1 channels in glomerulosclerosis has not been investigated so far.

The K_Ca_3.1 channel is voltage independent but gated by intracellular Ca^2+^ that binds to calmodulin, a Ca^2+^-binding protein that is constitutively associated with the C terminus of each channel subunit, and opens the channel [11]. Its inhibitors include two structurally distinct groups, peptidic and nonpeptidic [12]. Clotrimazole and its derivative triarylmethane (TRAM-34) belong to the later. TRAM-34 blocks the K_Ca_3.1 channel only when applied from inside via the interaction with the P-loop amino acid Thy250 and the S6 segment amino acid Val275 [13]. Due to the high specificity to K_Ca_3.1 channels, TRAM-34 is so far the best probe to study the roles of K_Ca_3.1 channels [14].

Transforming growth factor-β1 (TGF-β1) is a polypeptide member of the transforming growth factor β superfamily of cytokines and performs many cellular functions, such as the control of cell growth, cell proliferation, cell differentiation and apoptosis [15]. Many studies demonstrate that TGF-β1 is an important regulatory factor involved in the inflammatory damage and in the regulation of phenotype transdifferentiation of glomerular and tubular cells, and that the overexpression of TGF-β1 may lead to renal fibrosis [16]–[18]. On the surface of mesangial cells there is a distribution of TGF-β1 receptors [19], [20]. Our previous experiments showed that TGF-β1 might induce the premature senescence and cellular phenotype transformation of mesangial cells [21].

In this current study, we adopted TGF-β1 (2 ng/ml) and TGF-β1 (2 ng/ml) + TRAM-34 (16 nM) separately to stimulate rat mesangial cells for specified times from 0 min to 60 min in vitro, and assessed the changes in cell cycle, phenotype and proliferation by detecting the expression of α-smooth muscle actin (α-SMA), the specific marker of myofibroblast phenotypic transformation of mesangial cells [22], and fibroblast-specific protein-1 (FSP-1), the specific marker of differentiation and proliferation of active fibroblasts [23]. Our data demonstrate that targeted disruption of K_Ca_3.1 may inhibit TGF-β1-induced premature aging, myofibroblast-like phenotype transdifferentiation and proliferation of mesangial cells.

## Results

### K_Ca_3.1 is located in the cell membrane of mesangial cells

**Figure 1:**
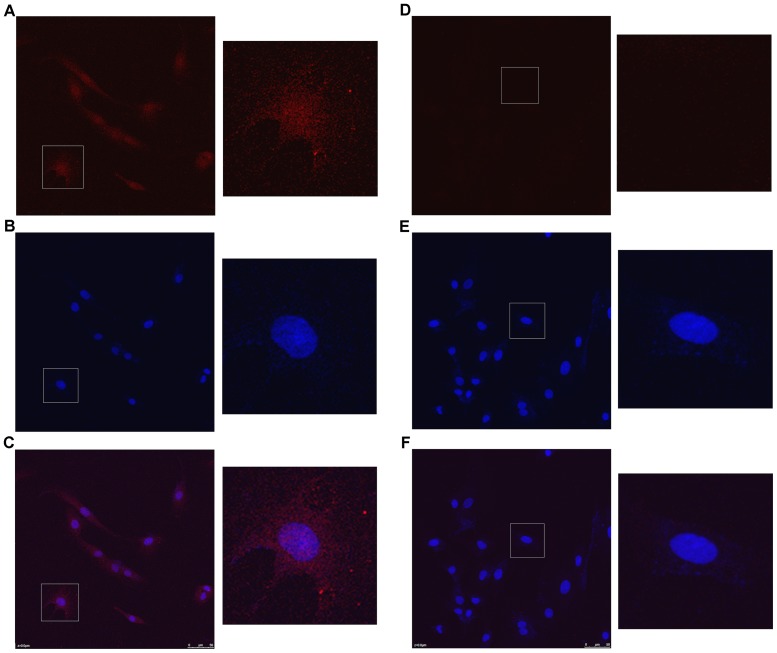
Confocal laser images of K_ca_3.1 channels in mesangial cell. (A) the cytoplasm image stained by the anti-K_ca_3.1 primary antibody and the Cy^Tm^3-conjugated Affinipure Goat Anti-Rabbit lgG (H+L) secondary antibody, (B) the nucleus image stained by DAPI and (C) the image overlaid by A and B. Correspondingly (D), (E), and (F) are the images of controls without the primary antibody.

Confocal laser images revealed that the K_ca_3.1 channels were distributed in the cell membranes and/or in the cytoplasm of mesangial cells **(Figure 1)**.

### TRAM-34 inhibits the TGF-β1-induced premature aging of mesangial cells

**Figure 2:**
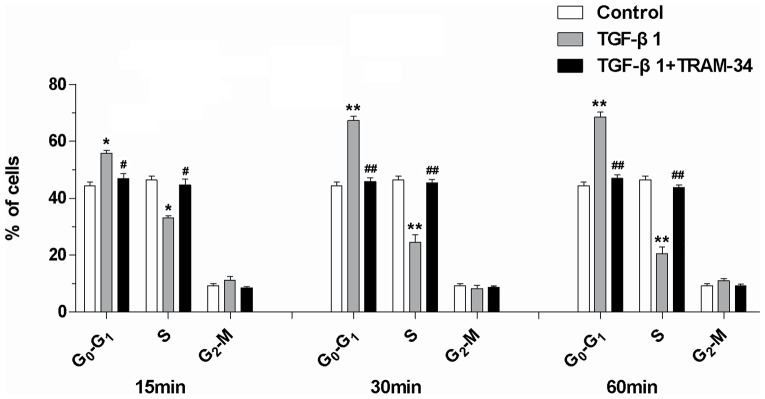
Changes in cell cycle progression at three time points under induction of TGF-β1 and TGF-β1+TRAM-34 (* P<0.05 and ** P<0.01versus control; ^#^ P<0.05 and ^##^ P<0.01 versus the TGF-β1-induced; n = 5).

The mesangial cells appeared to begin premature aging after 15 min stimulation of 2 ng/ml TGF-β1, presenting with significant increase in the percentage of cells in G_0_-G_1_ phase. And with the extension of stimulation time (30 min & 60 min), the percentage of cells in G_0_-G_1_ phase were gradually elevated, but the percentage of cells in S phase decreased **(Figure 2)**. Compared with the control, these changes were statistically significant (P<0.05), demonstrating that TGF-β1 induces premature aging of mesangial cells.

Under the combined stimulation of TGF-β1+TRAM-34, the percentage of mesangial cells in G_0_-G_1_ phase was obviously reduced and the percentage in S phase increased when compared with the TGF-β1-induced at three time points (P<0.05), and they approached to the control (**Figure 2)**, indicating that TRAM-34 may inhibit the TGF-β1-induced premature aging.

### TRAM-34 inhibits the TGF-β1-induced high expression of K_Ca_3.1 channels in mesangial cells

**Figure 3:**
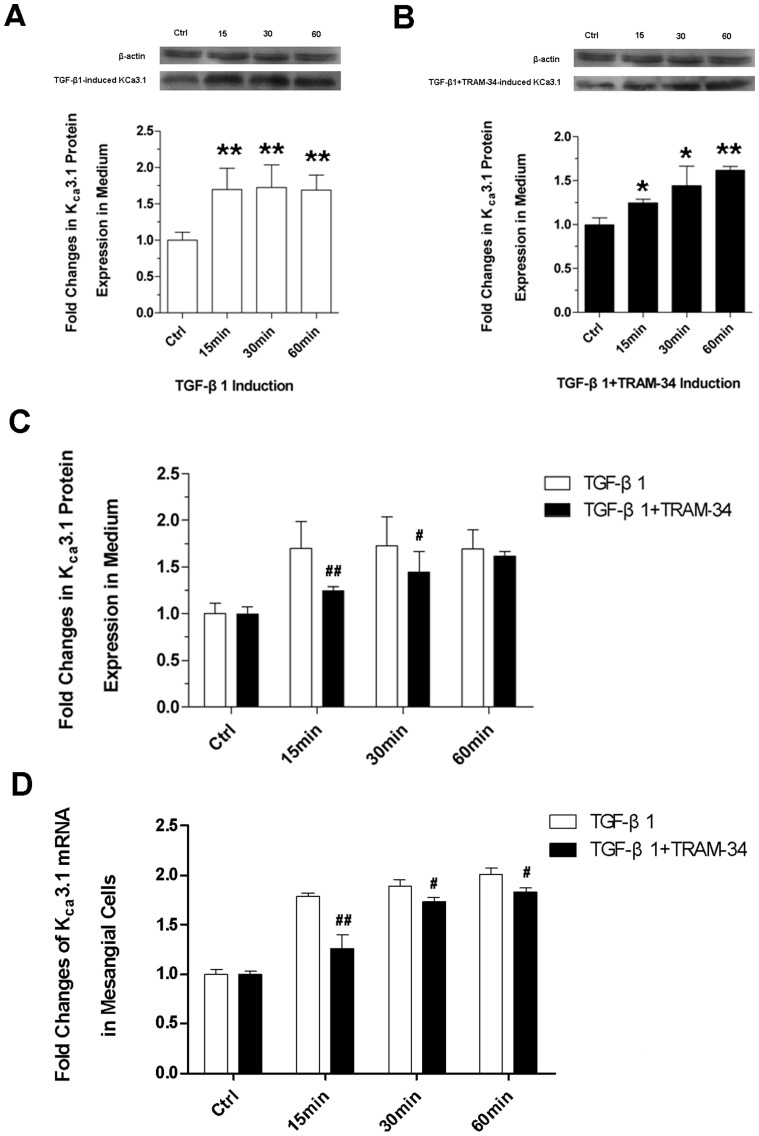
Expression of K_ca_3.1 channel. (A) Immunoblotting analysis of TGF-β1-induced K_ca_3.1 and (B) TGF-β1+TRAM-34-induced K_ca_3.1 protein expression with different induction time (15 min, 30 min, 60 min); (C) Differences between the TGF-β1- and the TGF-β1+TRAM-34-induced K_ca_3.1 protein expression at three time points; (D) RT-PCR assessments for K_ca_3.1 mRNA expression at three time points (* P<0.05 and ** P<0.01versus control; ^#^ P<0.05 and ^##^ P<0.01 versus the TGF-β1-induced; n = 3).

We used western blots to detect the protein expressions of K_Ca_3.1 in the cell lysates. It was found that compared with the control, TGF-β1-induced protein expression of K_Ca_3.1 was elevated at three time points (**Figure 3A)** (P<0.05 or P<0.01) and TGF-β1+TRAM-34-induced K_Ca_3.1 protein expression was also elevated since 30 min after stimulation **(Figure 3B)**. Although there was no statistical difference between the TGF-β1- and the TGF-β1+TRAM-34-induced K_Ca_3.1 protein expression at 60 min, the TGF-β1+TRAM-34-induced K_Ca_3.1 was significantly lower at 15 min and 30 min (P<0.05 or P<0.01) **(Figure 3C)**. Moreover in the subsequent mRNA detection, the TGF-β1+TRAM-34-induced K_Ca_3.1 mRNA expressions at three time points were all significantly lower than the TGF-β1-induced (P<0.05 or P<0.01) **(Figure 3D)**, indicating that TRAM-34 may inhibit the TGF-β1-induced high expression of KCa3.1 channels.

### TRAM-34 inhibits the TGF-β1-induced myofibroblast phenotypic transformation of mesangial cells

**Figure 4:**
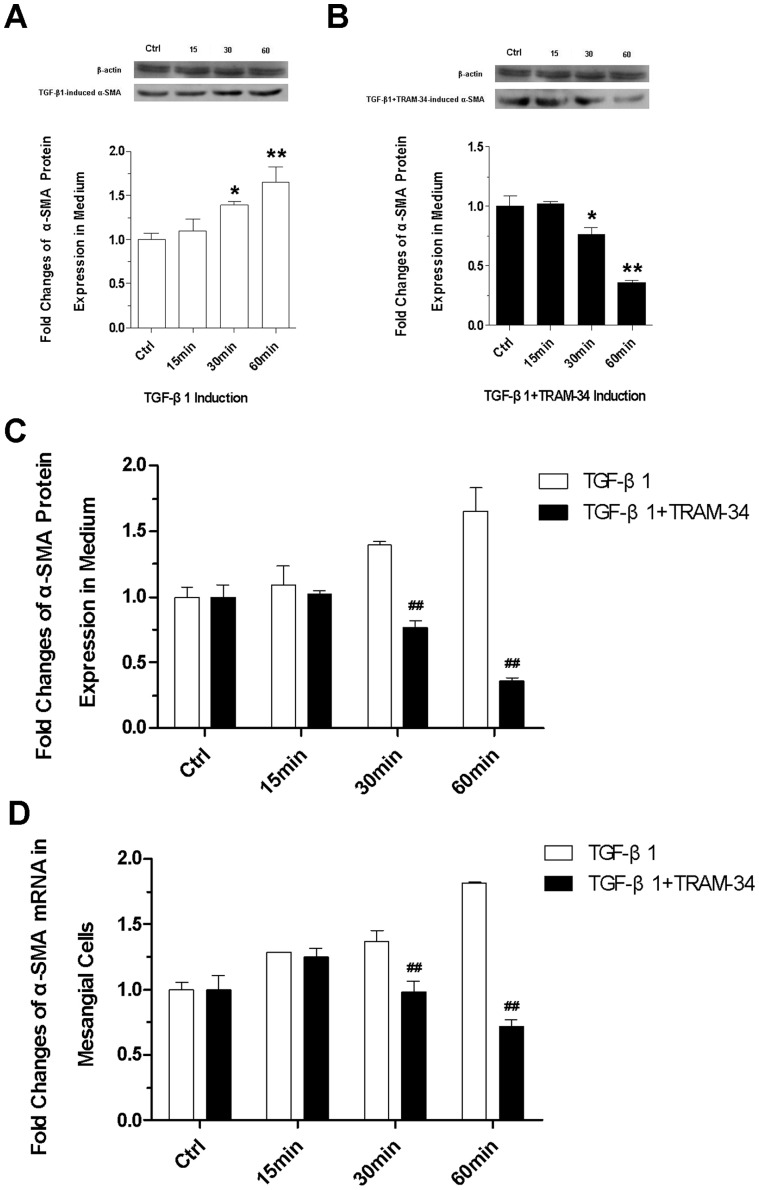
Expression of α-SMA. (A) Immunoblotting analysis of TGF-β1-induced α-SMA and (B) TGF-β1+TRAM-34-induced α-SMA protein expression with different induction time (15 min, 30 min, 60 min); (C) Differences between the TGF-β1- and the TGF-β1+TRAM-34-induced α-SMA protein expression at 30 min and 60 min; (D) RT-PCR assessments for α-SMA gene expression (* P<0.05 and ** P<0.01versus control; ^#^ P<0.05 and ^##^ P<0.01 versus the TGF-β1-induced; n = 4).

We detected α-SMA protein expression and its mRNA expression in mesangial cells. Western blot analysis showed that the TGF-β1-induced protein expression of α-SMA in the cell lysates was obviously higher and higher with induction time extension **(Figure 4A)**. Under the stimulation of TGF-β1+TRAM-34, α-SMA protein expression was slightly elevated at 15 min and then gradually decreased since 30 min after stimulation **(Figure 4B)** (P<0.05 or P<0.01). The differences between the TGF-β1- and the TGF-β1+TRAM-34-induced α-SMA protein expression were significant at 30 min and 60 min after stimulation **(Figure 4C)** (P<0.01). The detection of mRNA expression of α-SMA **(Figure 4D)** had the analogous findings, indicating that TRAM-34 may inhibit the TGF-β1-induced phenotype transition of mesangial cells.

### TRAM-34 inhibits the TGF-β1-induced fibroblastic proliferation of mesangial cells

**Figure 5:**
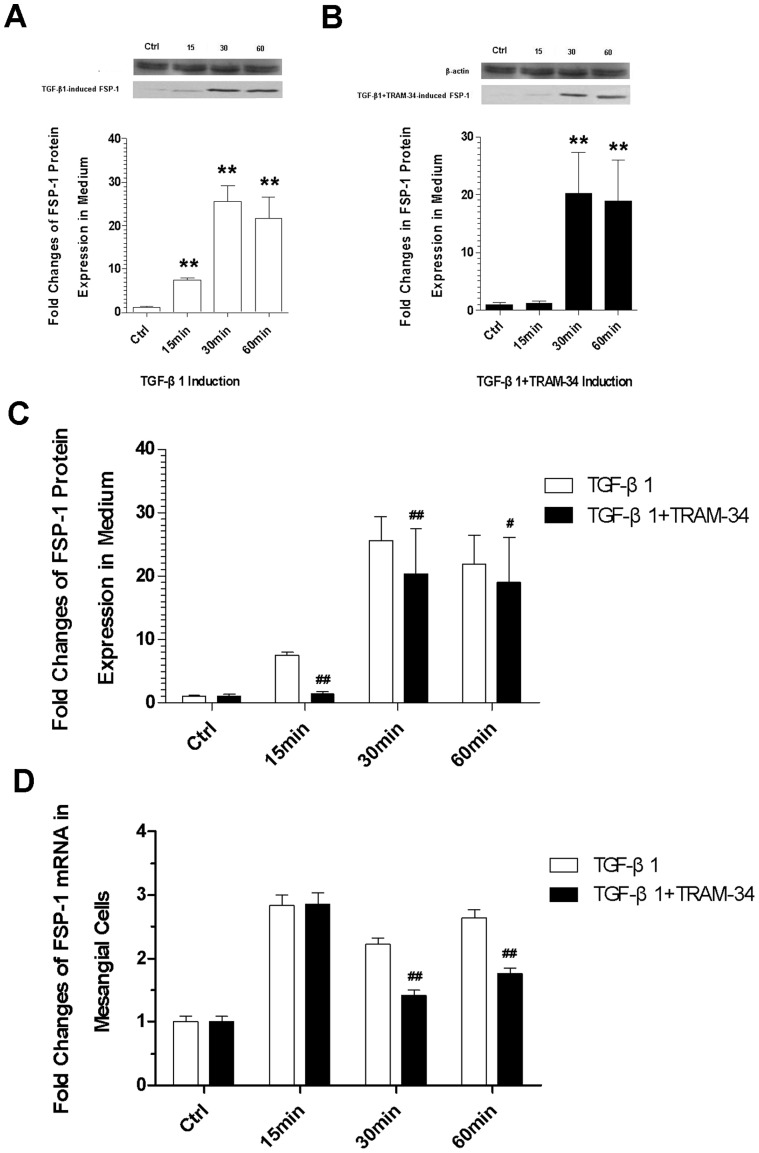
Expression of FSP-1. (A) Immunoblotting analysis of TGF-β1-induced FSP-1 protein expression; (B) Immunoblotting analysis of TGF-β1+TRAM-34-induced FSP-1 protein expression; (C) The differences between the TGF-β1- and the TGF-β1+TRAM-34-induced FSP-1 protein expression; (D) RT-PCR assessments for FSP-1 gene expression (* P<0.05 and ** P<0.01versus control; ^#^ P<0.05 and ^##^ P<0.01 versus the TGF-β1-induced; n = 5).

Under the stimulation of TGF-β1, the protein expression of FSP-1 was obviously increased at the three time points **(Figure 5A)** (P<0.01). Under the induction of TGF-β1+TRAM-34 the FSP-1 was also elevated at 30 min and 60 min after induction **(Figure 5B)** (P<0.01). In spite of that, the TGF-β1+TRAM-34-induced FSP-1 protein expressions measured at three time points were all lower than the TGF-β1-induced, pairwise comparison showed that the differences were statistically significant **(Figure 5C)** (P<0.05 or P<0.01). In addition, we found that the mRNA expressions of FSP-1 induced by TGF-β1+TRAM-34 were also obviously less than that induced by TGF-β1 at 30 min and 60 min **(Figure 5D)**. This means that TRAM-34 may inhibit the TGF-β1-induced proliferation of fibroblasts.

## Discussion

Pathological alteration of mesangial cells acts an important role in the development of glomerulosclerosis [24]. This current study investigated the potential involvement of K_Ca_3.1 channels of mesangial cells in the early stage of glomerulosclerosis. Our data suggest that inhibition of the K_Ca_3.1 channels reduces the TGF-β1-induced premature senescence, phenotype transition and proliferation of mesangial cells.

Based on current knowledge, resident fibroblast activation and proliferation in the kidney is triggered by locally secreted fibrogenic chemokines, including TGF-β1, PDGF, CTGF, and bFGF [10]. TGF-β1, a small molecular and soluble polypeptide, may potently promote proliferation of renal fibroblasts via a downstream mechanism that is largely mediated by bFGF [25], [26]. TGF-β1 has high affinity receptors on mesangial cells, and by binding to the receptors on mesangial cells, it can promote the secretion of mesangial matrix, type I and type IV collagen through ERK1/2, PI3K and JNK-MAPK and other signal pathways, contributing to the development of glomerulosclerosis [27], [28].

The effects of TGF-β1 on mesangial cells are bidirectional. Low concentrations of TGF-β1 (<100 pg/ml) promote cell proliferation, high concentrations (>250 pg/ml) inhibit mesangial cell proliferation and induces premature aging and/or hypertrophy [27], [28]. This current study substantiates it again and demonstrates that induction of high concentration of TGF-β1 (2 ng/ml) makes the cell cycle of mesangial cells arrested in G0/G1 phase, leading to the occurrence of premature senescence of mesangial cells.

K_Ca_3.1 channel is a member of the calcium-activated potassium channel (KCa) family and has the overall architecture of the voltage-gated K (Kv) channel super family, with four sub-units, each containing six transmembrane domains (S1–S6) and a pore domain (P loop) located between S5 and S6 [11]. K_ca_3.1 gene product contains 428 amino acids. Its N-terminal is located in the inner membrane and its C-terminal is also terminated at the inner membrane [29]. It has been confirmed that mesangial cells have a distribution of K_Ca_3.1 channels [30]. The confocal laser scanning in this study found that the Kca3.1 channels exist in the cell membranes and/or in the cytoplasm of mesangial cells. Basically this finding is consistent with the previous studies above.

Studies confirm that mitogens such as bFGF, PDGF, or VEGF may distinctly up-regulate KCa3.1 in several cell types [31], [32]. Congruent with this, our data display that TGF-β1 may obviously raise the expressions of K_Ca_3.1 in mesangial cells. TRAM-34 is the selective inhibitor of K_Ca_3.1 channel [13]. After adding the TRAM-34, the high expression of K_Ca_3.1 mRNA was significantly down-regulated at three time points. Recently Huang et al. showed that K_Ca_3.1 mediates renal fibrosis through the TGF-β1/Smad signaling pathway and TRAM34 can reduce TGF-β1-induced phosphorylation of Smad2/3 and ERK1/2 [33].

It is worth noting that although TRAM-34 has a certain inhibition effect to the TGF-β1-induced high expression of K_Ca_3.1, TRAM-34 can not fully inhibit the TGF-β1 induction effect. Related to the controls, the TGF-β1+TRAM-34-induced K_Ca_3.1 protein and mRNA expression were gradually elevated at three time points and at 60 min the inhibition to K_Ca_3.1 protein became weaker and insignificant. But the inhibition to K_Ca_3.1 mRNA expression was still significant. It is reliable in this study to ascertain the inhibition of TRAM-34 to the TGF-β1-induced K_Ca_3.1 high expression at early time points.

Our data show that TGF-β1 induces high expression of α-SMA in the mesangial cells. This finding is congruent with Stephenson's study. He and his colleagues found that α-SMA expression was up-regulated to at least 10 times higher 3-5 days after mesangial cells were co-cultured with 10 ng/ml TGF-β1 and meanwhile the cell volume of mesangial cells was enlarged. They thought that the expression of α-SMA reflected more the hypertrophy and hyperplasia of mesangial cells rather than the proliferation [28]. α-SMA is a mechanosensitive protein that can be rapidly recruited to β-cytoplasmic actin stress fibers under high tension [34]. It is generally considered to be the iconic antigen of mesangial cells in active state and hallmarks the myofibroblast phenotypic transformation [22]. High expression of α-SMA in this study means TGF-β1 may induce myofibroblast phenotype transition of mesangial cells.

Cell phenotype transdifferentiation is an important reaction of cells to injury, it can lead to serious pathological states, such as vasculitis [35], chronic rejection after renal transplantation [36], diabetic nephropathy [37]–[39] and acute tubular injury caused by ischemia/reperfusion [40]. In a pathological state, mesangial cells may swell and transform into myofibroblasts that express the specific phenotypic marker α-SMA and meanwhile produce collagen (type I and type IV), glycoproteins (fiber connexin, laminin and actin) and proteoglycans in a large quantity. In addition, the myofibroblast-like phenotype transformed cells may secrete matrix metalloproteinase inhibitors to degrade the activity of matrix metalloproteinase, leading to the generation of mesangial extracellular matrix greater than its degradation and the excessive sedimentation in renal interstitium [22]. A previous study indicates that the mesangial cells with the expression of α-SMA have a strong contraction capacity and this may result in renal structure remodeling and glomerular ischemic sclerosis [27].

In this study, FSP-1 is also highly expressed in the mesangial cells under TGF-β1 induction. FSP-1, member of S100 gene family, is also called S100A4 protein that is expressed in the cytoplasm of fibroblasts [41]. FSP-1 is one of cytoskeletal proteins and closely related to the microtubule dynamics, signal transduction, cell cycle regulation, cell growth and differentiation [41]. Nishitani et al. confirmed that FSP-1 was not detected in normal mesangial cells, tubular cells and endothelial cells, but in fibroblasts and fibrosis of the kidney FSP-1 had high expression. They thought FSP-1 was the specific marker of differentiation and proliferation of active fibroblasts [23]. So we think the expression of FSP-1 in this study may indicate the presence of a molecular program determining fibroblast phenotype transdifferentiation and proliferation of mesangial cells under induction of TGF-β1.

We think that K_Ca_3.1 channels participate in the process mediated by TGF-β1 that induces premature senescence, phenotype transition and proliferation of mesangial cells. Because our data demonstrates that when α-SMA and FSP-1 gene expression are increased under the induction of TGF-β1, K_Ca_3.1 gene expression is also elevated in the mesangial cells. After the K_Ca_3.1 channels are blocked using the specific inhibitor TRAM-34, the gene expression of K_Ca_3.1, α-SMA and FSP-1 are all decreased compared to the TGF-β1 induced group. This is in line with previous studies proving that KCa3.1 is highly expressed in a variety of proliferating cells [42], including smooth muscle cells, endothelial cells, lymphocytes (B- and T-cells), fibroblasts, stem cells and several cancer cells, where they participate in important cell functions, such as cell cycle progression, migration, and epithelial transport, by controlling the cell volume and the driving force for Ca^2+^ influx [42]–[44]. And this, like the increasing evidence, may suggest that KCa3.1 may play a pivotal role in disease states characterized by excessive cell proliferation [31], [44]–[46].

This current study focused on the early stage in which K_Ca_3.1 channels were involved in glomerulosclerosis. So we only observed the changes in KCa3.1, α-SMA and FSP-1 expression within 1 h. The observation time is short, which is the limitation of this study. Another limitation is that we didn't make correlation analysis to the relation of KCa3.1 with α-SMA and FSP-1 expression. Coupled with no in-vivo data, this study should be seen as preliminary exploration. In spite of that, we think that our data may indicate that targeted disruption of K_Ca_3.1 inhibits TGF-β1-induced premature aging, myofibroblast-like phenotype transdifferentiation and proliferation of mesangial cells.

## Materials and Methods

### Reagents

The main reagents used in this study included: DMEM/F12 (1∶1) (Gibco, USA); fetal bovine serum (FBS) (Hyclon, USA); MTT (Amresco, USA); EDTA, trypsin and DMSO (Sigma, USA); Recombinant human TGF-β1 (R & D System, USA); TRAM-34 (Biomol, product No.: BML-KC161-0005, 5 mg).

### Cell line & Cell culture

A cell line of rat mesangial cells (HBZY-1) purchased from the Type Culture Collection of the Chinese Academy of Sciences (Shanghai, China) was maintained in growth media (DMEM with 10% FCS, 2% penicillin/streptomycin). The cells (1×10^5^) in the log phase were switched to serum-free quiescent medium 48 h before stimulation to induce growth arrest.

### Stimulation

48 h after the cells (1×10^5^) were transferred to the serum-free quiescent medium, the quiescent medium was separately replaced by serum-free DMEM with TGF-β1 (2 ng/ml) and TGF-β1 (2 ng/ml) in combination with TRAM-34 (16 nM). Then the cells were sent to incubation in a humidified 5% CO_2_ incubator at 37°C for 0 min, 15 min, 30 min and 60 min respectively. The cells without treatment served as the controls.

### K_Ca_3.1 positioning

Confocal laser scanning microscope (Leica) was used to locate KCa3.1 channels in mesangial cells. Briefly, the procedure was: 48 h culture of the cells that grew to 70% confluence on cover slips, PBS washes, 10 min fixation in the chilled 4% formaldehyde, PBS washes, 30 min blocking in PBS with 3% BSA, 1 h incubation at room temperature with the anti-K_ca_3.1 primary antibody (diluted to 1∶200 in PBS with 3% BSA), PBS washes, 1 h incubation with Cy3-AffiniPure F(ab')2 Fragment Goat anti-Rabbit secondary antibody, PBS washes, 5 min DAPI staining, PBS washes, and capturing images with confocal laser scanning microscope.

### Cell cycle analysis

After the stimulations, the cells were digested with 0.25% trypsin. The cell suspension was collected, centrifuged (1000 rpm, 7 min) and washed with PBS, and then the supernatant was discarded. The cells were fixed in 70% precooling ethanol (1-ml) for 1∼2 h at 4°C. After PBS washes, the cells were treated with RNase (50 µg/ml) and sent to incubation at 37°C for 30 min. Centrifugation (1500 rpm, 5 min) and discarding supernatant were followed by that the cells were re-suspended in PBS containing propidium iodide (0.05 mg/ml in 3.8 mol/l natrium citrate; Labest Biotechnology Co., Ltd., Beijing) at room temperature for 30 min. Then, the stained cells were analyzed using flow cytometry (FACS Caliber, Becton-Dickinson, San Jose, CA, USA) according to the manufacturer's instructions and the data stored as listmode files. DNA cell cycle histograms were analyzed and modeled using ModFit and WinList software (Verity Software House, Topsham, ME, USA). Twenty thousand cells were analyzed in triplicate for each sample.

### Western blot analysis

15 min, 30 min and 60 min after stimulation the cells were washed twice with cold PBS and were incubated with cold lysis buffer (50 mM Tris/HCl [pH, 7.4], 150 mM of NaCl, 1 mM of EDTA, 1% Triton X-100, 1% sodium deoxycholate, and 1% Nonidet-P40, 0.1% sodium dodecyl sulfate [SDS]) supplemented with 0.1 mM DTT, protease inhibitor (Roche applied science, Mannheim, Germany) on ice for 30 min. Cellular debris was removed by centrifugation at 12,000 rpm for 20 min at 4°C. The total protein concentration was detected using a BCA Protein Assay Kit (Thermo Fisher Scientific, Rockford, USA). The samples were mixed with loading buffer and boiled for 5 min for denaturing. For immunoblotting analysis, 30 µg proteins were assayed using a 10% SDS-PAGE gel (150 V, 70 min) and transferred to a PVDF membrane (Immobilon-P™, Millipore) (350 mA, 3 h, 4°C) and blocked with PBS containing 5% skimmed milk at room temperature for 2 h. Subsequently, the primary antibody of α-SMA (1∶300), K_ca_3.1 (1∶300), FSP-1 (1∶100) and β-actin (1∶100) was added to the samples respectively and kept over night at 4°C. After 2 h incubation with the horseradish peroxidase-labeled goat anti-rabbit IgG secondary antibody (1∶100,000, Jackson ImmunoResearch, USA), the samples were color-developed using ECL reagent (Pierce, USA) and imaged. The images were scanned and analyzed using ImageJ.

### Real Time RT-PCR

Total RNA of the cells after stimulation was extracted using Trizol Kit (Tiangen Biotech (Beijing) Co., Ltd). RNA purity and content were determined with UV/visible spectrophotometer. Synthesis of cDNA was conducted with TaKaRa reverse transcription kit (PrimeScriptII1st Strand cDNA Synthesis Kit) according to the manufacturer's instructions. After reverse transcription, the resulting materials were used for PCR amplification using gene-specific primer pairs and RealMasterMix (SYBR Green) kit (Tiangen Biotech (Beijing) Co., Ltd., Beijing, China). α-SMA, K_ca_3.1, FSP-1 and GAPDH mRNA were determined quantitatively by using Thermal Cyeler Dice Real Time System (TP800, Takara, Japan) and GAPDH as intra-contrast gene or internal control. All steps were performed on ice.

The sequences of α-SMA, Kca3.1, FSP-1 and GAPDH are shown in **Table 1** and the amplification conditions for real-time PCR were: initial denaturation (95°C, 3 min), 40 cycles of denaturation (95°C, 10 sec), annealing (60.5°C, 10 sec), extension (72°C, 10 sec), and then a final extension (72°C, 10 min).

**Table 1:** The sequences of Kca3.1, α-SMA, FSP-1 and GAPDH.

	*Sequence*	*Product Length (bp)*
***Kca3.1***	5′TGGAGCTGCTGGTATGCG3′	228
	3′ACCTCGACGACCATACGC5′	
***a-SMA***	5′GGAGAAGCCCAGCCAGTCGC3′	115
	3′CCCGCCTTACAGAGCCCGGA5′	
***FSP-1***	5′ATGTAATAGTGTCCACCTTCC3′	181
	3′ACTTCATTGTCCCTGTTGCT5′	
***GAPDH***	5′GCAAGTTCAACGGCACAG3′	140
	3′CGTTCAAGTTGCCGTGTC5′	

### Statistics

Experiments were repeated at least three times, and the number of repetitions is represented in the figure legends by “n = ”. Data are expressed as the mean ± SEM. Group differences were analyzed with one-way analysis of variance (ANOVA) and pairwise comparison with Student-Newman-Keuls-q test (SNK-q). Statistical significance was considered at P<0.05. All analyses used the statistical package SPSS for Windows 13.0.
